# Systemic Sclerosis, Reversible Cerebral Vasoconstriction Syndrome, and NeuroMyelitis Optica in a Patient

**DOI:** 10.1155/2022/8541329

**Published:** 2022-07-12

**Authors:** Masoud Etemadifar, Mehdi Shafiei, Mehri Salari, Ali Modares Sadeghi, Mohammad Fakhrolmobasheri

**Affiliations:** ^1^Department of Neurology, Isfahan University of Medical Sciences, Isfahan, Iran; ^2^Department of Neurosurgery, Isfahan University of Medical Sciences, Isfahan, Iran; ^3^Functional Neurosurgery Research Center, Shohada Tajrish Comprehensive Neurosurgical Center of Excellence, Shahid Beheshti University of Medical Sciences, Tehran, Iran; ^4^Isfahan Cardiovascular Research Center (Heart Failure Research Center), Isfahan, Iran

## Abstract

Systemic sclerosis (SSC) is an autoimmune disease of connective tissue and microvasculature mostly caused by autoantibodies. Likewise, neuromyelitis optica (NMO) is a demyelinating disease of the central nervous system correlating with autoantibodies against aquapourin-4. Reversible cerebral vasoconstriction syndrome (RCVS) is a disorder of brain vasculature resembling Raynaud phenomena in SSC. Despite co-occurrence is not rare in autoimmune disorders, the co-occurrence of NMO and SSC is extremely rare. In this case, we report a 35-year-old female presenting with paraplegia one day after discharge from hospital following surgical carnioplasty. She had a history of scleroderma and optic neuritis for which she was treated with high dose glucocorticoids causing renal crisis and RCVS causing intracranial and intracerebral hemorrhage which required a craniotomy to be performed in February 2020. In her recent admission, magnetic resonance imaging of the spinal cord indicated longitudinally extensive transverse myelitis (LETM) and blood tests revealed a highly positive titer of NMO-IgG. Daily plasmapheresis resulted in satisfactory improvement in her condition. This case highlights the importance of evaluating neurologic manifestations in systemic sclerosis patients considering the NMO and RCVS occurrence. Additionally, in concomitant cases, the treatment strategy should be modified regarding the risk of scleroderma renal crisis.

## 1. Introduction

Systemic sclerosis (SSC) is a connective tissue disorder with organ specific manifestations limited to the skin or systemic manifestations including lungs, kidneys, and gastrointestinal system involvement. NMO is characterized by excessive stromal collagen deposition and autoimmunity caused by autoantibodies which are mostly reported to be *anti*-topoisomerase, *anti*-scl70, and *anti*-nuclear antibody. Microvascular complications in SSC may manifest as Raynaud phenomena, renal crisis, and pulmonary hypertension [1]. Raynaud phenomena could also appear in the brain causing reversible cerebral vasoconstriction syndrome (RCVS) [2]. RCVS is a neuro-vascular disorder characterized by reversible spasm of cerebral arteries [3]. The exact pathophysiology of RCVS is not clearly defined; however, several risk factors are discovered including the administration of high dose glucocorticoid [4]. RCVS may contribute to subarachnoid [5] and intracerebral hemorrhages [6] which may contribute to major morbidity for patients. The incidence of RCVS in the context of autoimmune disorders is rarely reported [7, 8].

Renal crisis is a lethal complication of systemic sclerosis manifesting with malignant hypertension and acute renal failure. This condition may occur in patients treated by high dose glucocorticoids and could pose permanent damage to the kidneys [9].

Neuromyelitis optica (NMO) is an autoimmune disease of the CNS mostly involving the optic nerves, spinal cord, and brainstem mainly manifesting with blurred vision, acute onset paralysis, intractable nausea, vomiting, and hiccups. Antibodies against aquapourin-4, the prime channel regulating cellular hemostasis in the CNS, are the main antibodies discovered in association with NMO (NMO-IgG) [10]. The autoantibodies induce necroptosis in myelin producing cell manifesting as inflammatory demyelination of the involved region in the CNS. The relapsing behavior of NMO may eventually lead to extreme morbidities for the patient whereas severe optic neuritis may lead to blindness, longitudinal extensive transverse myelitis (LETM) may lead to paralysis, and brainstem involvements could end in acute central respiratory failure [11].

As any other autoimmune diseases, NMO is reported in association with many systemic autoimmunities such as systemic lupus erythematous, sarcoidosis, and Sjögren's syndrome. NMO is also associated with organ specific autoimmune diseases such as autoimmune thyroiditis, myasthenia gravis, idiopathic thrombocytopenic purpura, and ulcerative colitis. However, the coexistence of NMO and systemic sclerosis and further complications in treatment is rarely reported [10, 12]. On the other hand, the occurrence of RCVS in the context of SSC is explainable through similar mechanisms but is difficult to correlate.

Herein, we present a patient with systemic sclerosis who later developed optic neuritis. She was treated for optic neuritis with glucocorticoids which targeted scleroderma renal crisis and RCVS. The RCVS further caused SAH. Regarding increased ICP, she underwent a craniotomy surgery. Two months later, after resolution of increased ICP, carnioplasty was performed for her. One day after discharge from the neurosurgery ward, she presented again with paraplegia and urinary retention. Further investigations revealed LETM which was consistent with NMO.

## 2. Case Report

A 35-year-old female referred to our clinic complaining of paraplegia and urinary retention one day after discharge from the hospital following a carnioplasty surgery. Her medical history revealed that 2 years ago she had been diagnosed with systemic sclerosis manifested as intractable nausea and vomiting. At that time, routine workups had been insignificant except for a history of chronic fatigue and hypothyroidism. The hypothyroidism was diagnosed 1 year before the diagnosis of SSC. The patient's records indicated that the thyroid dysfunction was diagnosed to be due to Hashimoto thyroiditis with positive *anti*-thyroid peroxidase antibodies (*anti*-TPO). Physical examination was indicative for cutaneous manifestations of scleroderma; Rodnan score 2 (puffy hands and shiny skin with skin thickening and a dull face). In addition, high erythrocyte sedimentation rate (ESR) in laboratory studies and a grade II esophagitis in upper GI endoscopy was noted in her medical records. After a comprehensive rheumatologic workup, the diagnosis of systemic sclerosis was eventually confirmed. The laboratory records indicated *anti*-nuclear antibody = 1/3200 (positive >1 : 100) with a nuclear large/coarse speckeld pattern, *anti*-nRNP/sm = 92 (strong positive >50), *anti*-sm = 8 (6 < borderline < 10), *anti*-nucleosomes antibody = 20 (11 < positive+ < 25), *anti*-histones = 20 (11 < positive+ < 25), and *anti*-cyclic citrollinated peptide antibody = 29.3 (positive >18). The diagnosis was made according to ACR/EULAR criteria for classification of systemic sclerosis with a score of 15 (skin thickening = 9, abnormal nailfold capillaries = 2, Raynaud phenomenon = 3, laboratory findings = 1) [13]. The patient was treated with calcium channel blockers, proton pump inhibitors, and fluoxetine. For about one and a half years, her clinical status was stable. About 6 months later, she experienced blurred vision in her left eye. MRI of the brain was normal; however, optic perimetry indicated an altitudinal field defect in the left. Ophthalmology consultation proposed the diagnosis of optic neuritis in association with systemic sclerosis. Thus, a methylprednisolone pulse was administered in four consecutive doses of 1 gram daily under close observation. 24 hours after administration of the last dose, she experienced a sudden onset of thunderclap headache, nausea, vomiting, and loss of consciousness. Early assessments revealed a blood pressure of 220/190, GCS of 7/15, reactive and symmetric pupils, no gaze, and positive corneal and gag reflex. Brain computed tomography (CT) implied huge intracranial hemorrhage in the left parietotemporal region. Cerebral arteries angiography showed severe vasospasm in both internal carotid arteries at c4 and c6 segments and left middle cerebral artery's M1 which is consistent with reversible cerebrovascular spasm (RCVS). Eventually, considering very high intracranial pressure, she underwent an extensive craniotomy for brain decompression, and after a month of hospitalization, she was discharged with GCS = 15, left hemiplegia, and dysarthria. After 4 months of observation, the patient's status became eligible for cranioplasty. She underwent the surgery and after 4 days of hospitalization, she was discharged with a favorable clinical status.

**Figure 1:**
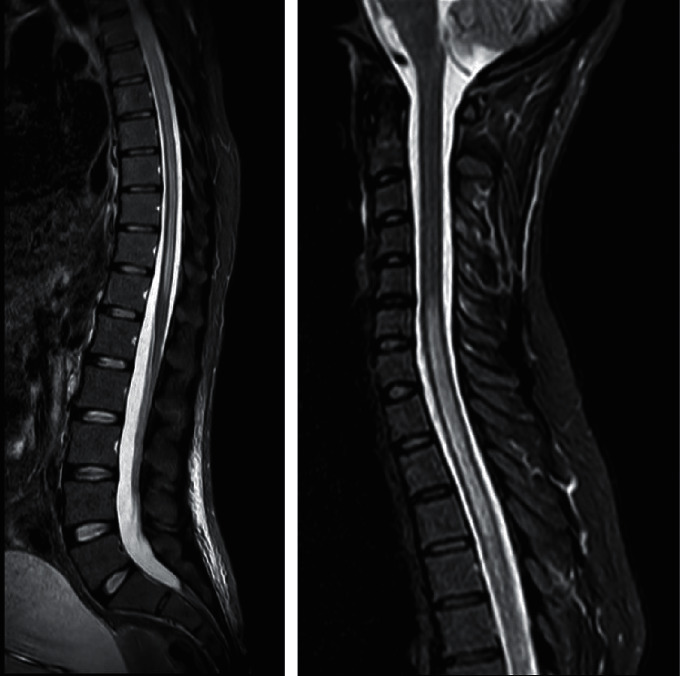
STIR view in magnetic resonance imaging of the patient's spinal cord indicates longitudinally extensive transverse myelitis.

24 hours later, on February 18, 2021, she was readmitted to our clinic with complaints of new onset paraplegia and urinary retention which could have been easily misdiagnosed for postoperative complications. On the contrary, physical examinations demonstrated bilateral positive Babinski sign and a sensory level at C4 which led us to suspect a new onset spinal disorder rather than any adverse incidence following the cranioplasty. Therefore, we performed a magnetic resonance imaging (MRI) of the spinal cord, and the results showed a longitudinally extensive transverse myelitis (LETM) from C4 to conus medullaris (Figure 1). The blood test results indicated high titer of *anti*-aquaporin4 antibody (1 : 320) which confirmed the diagnosis of NMO. Consequently, she underwent plasmapheresis (for 7 days, 250 ml/kg) without methylprednisolone. Following the treatment, her clinical status started to improve and the new onset paralysis in her left leg gradually diminished. After 9 days of hospitalization, the patient was discharged with a favorable clinical status and the ability to walk with minor assistance.

### 2.1. Ethical Statement

This study was approved by committee of research, neurosurgery department, Isfahan University of Medical Sciences, Iran. Written informed consent was obtained from the patient. The consent form is available on reasonable request to the corresponding author.

## 3. Discussion and Conclusion

Systemic sclerosis is rarely reported in association with NMO. According to the study by Bollo et al., till now, there has been only seven cases reported to be suffering from concomitant NMO and SSC in which all of them were treated by glucocorticoids [14]. The time of onset of SSC and NMO differed among cases as in the case reported by Hernandez et al. NMO was developed years after the diagnosis of SSC and in the case studied by Moriguchi et al. NMO was diagnosed one year before the symptoms of SSC. Interestingly, all cases were female with age from 30 to 65 years. None of the reported cases had concomitant thyroid disorder except in our case who was diagnosed for hypothyroidism about 3 years before the diagnosis of NMO. Thyroid disorders such as Hashimoto thyroiditis may accompany NMO. Shahmohammadi et al. had postulated that autoimmune thyroiditis, Hashimoto thyroiditis, and graves' disease are the most common organ specific autoimmune disorders associated with NMO. The presence of *anti*thyroid antibodies is frequently reported in patients with NMO. Moreover, it has been reported that thyroid follicular cells may have AQP4 containing channels [15]. In another review by Fallahi et al., the association between thyroid autoimmunity and SSC is extensively studied. The authors postulated that T-helper 1 immune predominance in combination with vitamin D deficiency may be the same triggers for autoimmune thyroid disorders and SSC [16]. Nevertheless, the coexistence of thyroid autoimmunity, SSC and NMO in the present case is novel in the literature and may be a clue for further researches in this field whereas the treatment of such conditions may become challenging due to the fact that glucocorticoid treatment in SSC patients may trigger the development of renal crisis. Renal crisis may manifest as hypertension and acute renal injury. Although the condition satisfactorily responds to treatment with angiotensin converting enzyme inhibitors (ACEI), severe complications such as malignant hypertension, intracranial hemorrhage, and permanent renal impairment could still ensue [9]. In the case reported by Deeb et al. [17], the patient developed systemic sclerosis renal crisis following the administration of glucocorticoid; however, other cases of which all were treated with high dose methylprednisolone did not develop renal crisis [12, 14, 18–21]. Accordingly, in the present case, intracerebral and subarachnoid hemorrhage after administration of glucocorticoids was notable in her medical history.

Another interesting finding from the studies is that some cases did not respond well to the treatment with steroids and the patients were subsequently treated with other immunosuppressive agents such as cyclophosphamide [14]. This might highlight the importance of choosing proper treatment for patients with such complicated condition. Another point to consider is that the diagnosis of RCVS during cerebral angiography could raise the doubt whether the ICH and SAH were complications of RCVS or the combination of hypertension and RCVS caused the stroke [3, 9]. Was stroke a complication of renal crisis and hypertension or RCVS or both? In all scenarios, it is clear that the high dose glucocorticoid administration triggered the event.

This case and other cases reporting the association of systemic sclerosis with NMO could improve our knowledge to evaluate SSC patients with neurologic disorders. Symptoms indicative of optic neuritis, transverse myelitis, and brainstem involvement may need a comprehensive workup including measurement of the serum levels of NMO-IgG and MRI of the spinal cord. It is also remarkable that according to the guidelines of the treatment of NMO [22], the first line therapy in NMO flares is high dose glucocorticoids; however, glucocorticoid administration in patients with systemic sclerosis is not considered the best choice due to the risk of renal crisis. Thus, the controversy rises surrounding the treatment of NMO patients with systemic sclerosis. As previously mentioned, the risk of renal crisis in SSC patients is not very high, but it may cause severe morbidities as reported in our case. Moreover, to our knowledge, RCVS in systemic sclerosis is not reported, but it could be a complication of SSC regarding the similarity of mechanism with Raynaud phenomenon.

In conclusion, neurologic manifestations in patients with systemic sclerosis are better to be evaluated precisely to rule out NMO using NMO specific IgG testing. Eventually, if the diagnosis of NMO is confirmed, then the treatment strategy may be modified using plasmapheresis in combination with other immunosuppressive drugs such as rituximab and methotrexate to reduce the risk of development of renal crisis.

## Data Availability

All data are available upon reasonable request to the corresponding author.
